# Novel heterozygous *F7* gene mutation (c. C1286T) associated with congenital factor VII deficiency: A case report and literature review

**DOI:** 10.1002/jcla.24349

**Published:** 2022-03-29

**Authors:** Hua Tang, Xingzhao Luan, Jiaqi Li, Gen Jiang, Haowen Zhen, Hao Li, Wei Xiang, Jie Zhou

**Affiliations:** ^1^ Department of Neurosurgery the Affiliated Hospital of Southwest Medical University Lu Zhou China; ^2^ 74647 Southwest Medical University Lu Zhou China; ^3^ Sichuan Clinical Research Center for Neurosurgery Lu Zhou China; ^4^ Academician (Expert) Workstation of Sichuan Province Lu Zhou China; ^5^ Neurological Diseases and Brain Function Laboratory Lu Zhou China

**Keywords:** congenital factor VII deficiency, intracranial hemorrhage, mutation

## Abstract

**Background:**

Congenital factor VII (FVII) deficiency is a rare inherited autosomal recessive disorder characterized by prolongation of prothrombin time and low FVII coagulation activity, which may increase the risk of bleeding.

**Case presentation:**

A 66‐year‐old man with acute postoperative intracranial hemorrhage was transferred to our hospital owing to coagulation dysfunction. In coagulation tests, the FVII coagulation activity was less than 2%. Genetic analysis of the gene encoding FVII identified compound heterozygous mutations: c. 681+1 G>T and c. C1286T (p. Ala429Val).

**Conclusions:**

To our knowledge, this is the first report describing the c. C1286T (p. Ala429Val) mutation in the FVII‐encoding gene. We suggest that these mutations resulted in the reduced FVII activity and abnormal clotting in our patient after brain surgery.

## Graphical abstract

Computed tomography (CT) scan showed postoperative rebleeding at the right basal ganglia.

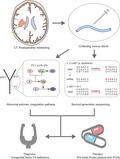

## 1 INTRODUCTION

Congenital factor VII (FVII) deficiency (OMIM: 227500) is a rare autosomal recessive bleeding disease defined by FVII activity less than 70% of normal.^1^ Its estimated prevalence is 1/500,000,^2^, ^3^ and its clinical manifestations range from asymptomatic to severe or even fatal bleeding.

FVII is a coagulation factor and serine protease; when active, it initiates the extrinsic coagulation pathway. The percentage of FVII coagulation activity (FVII:C) determines whether FVII deficiency is mild (>20% but <70%), moderate (10–20%), or severe (<10%).^4^ Mild FVII deficiency is usually asymptomatic and is diagnosed incidentally during pregnancy, delivery, or preoperative examinations or by prolonged bleeding due to surgery or post‐traumatic events.^5^ Moderate FVII deficiency usually occurs during puberty, especially in women at menarche. Severe FVII deficiency usually occurs at an early age and is serious and life‐threatening. Despite various therapeutic options, including administration of fresh frozen plasma, recombinant activated FVIIa (rFVIIa), prothrombin complex concentrate, or plasma‐derived FVII concentrate, the treatment of FVII remains difficult owing to the scarcity of information regarding its clinical management.

## 2 CASE PRESENTATION

**FIGURE 1:**
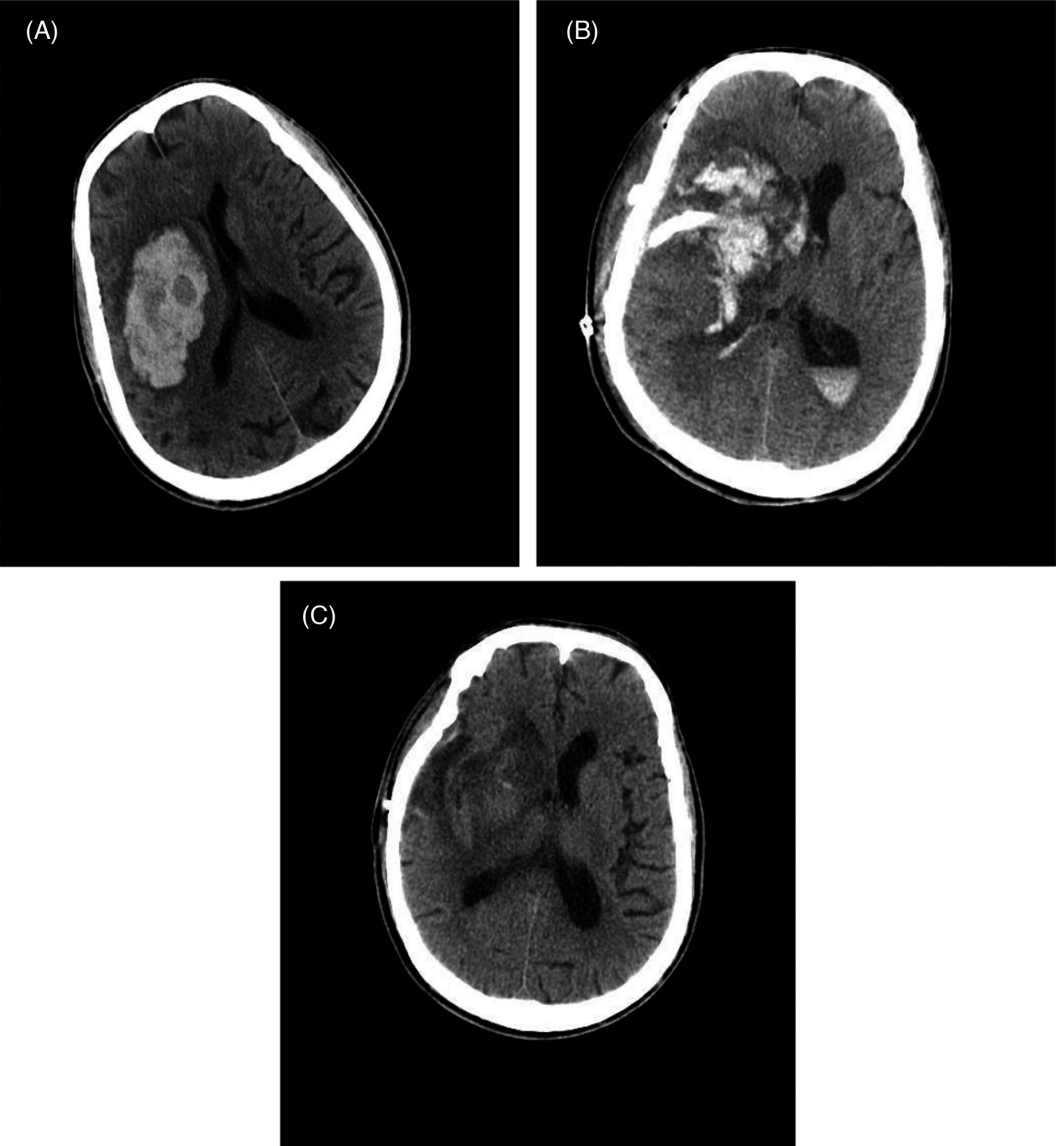
Cranial CT examination. Cranial (A), (B), and (C) CT images of the head showed preoperative, admission, and discharge intracranial conditions, respectively

A 66‐year‐old man with acute postoperative intracranial hemorrhage was referred to our hospital owing to coagulation dysfunction. His Glasgow Coma Scale score was E1VTM2. Laboratory examination revealed a significantly isolated prolonged prothrombin time (PT) (33.6 s) and low FVII coagulation activity (1.8%); the activated partial thromboplastin time and thrombin time were normal. The patient did not take anticoagulants or antiplatelet drugs and had no history of bleeding diseases. We collected and collated the relevant examination reports of this patient, and the results are shown in Figure 1 and Table 1.

**TABLE 1:** Coagulation screening tests

Test	Result at admission	Result at discharge	Reference	Unit
PT	33.6(↑)	14.2(↑)	9.8–12.1	S
APTT	22.6	25.9	22.3–32.5	S
TT	16	15.4	14–21	S
Fib	3.86(↑)	3.66(↑)	1.8–3.5	g/L
II	108.1	107.4	70–120	%
VII	1.8(↓)	40.1(↓)	70–120	%
X	114.8	112.8	70–120	%
VIII	198.6(↑)	194.4(↑)	70–150	%
IX	143.5(↑)	151.5(↑)	70–120	%
XI	124.6(↑)	130.6(↑)	70–120	%
ALT	10.2	16.4	9–50	U/L
AST	18.6	25.6	15–40	U/L
PLT	184	212.4	125–350	10^9^/L
MPV	10.10	10.80	9.4–12.5	Fl
PCT	0.19	0.20	0.10–0.28	‐

Abbreviations: PT, prothrombin time; APTT, Activated partial thrombin time; TT, thrombin time; Fib, fibrinogen; II, blood coagulation factor II; VII, blood coagulation factor VII; X, blood coagulation factor X; VIII, blood coagulation factor VIII; IX, blood coagulation factor IX; XI, blood coagulation factor XI; ALT, alanine transaminase; AST, aspartate aminotransferase; PLT, Platelet count; MPV, mean platelet volume.

Because FVII deficiency is rare, we initially considered the following as potential causes of our patient's coagulation disorder. First, most clotting factors (except for factor III) are synthesized in the liver, and our patient had a history of hepatitis B. However, examination of transaminase levels indicated normal liver function (alanine transaminase, 10.2 U/L and aspartate transaminase, 18.6 U/L). Second, activation of the clotting factors (factors II, VII, IX, and X) is vitamin K‐dependent. Our patient had received vitamin K supplements at another hospital and thus was not vitamin K‐deficient. Third, although platelets are required for coagulation, routine blood tests revealed no abnormalities in platelet number or size (platelets, 184 × 10^9^/L; mean platelet volume, 10.10 fL; and platelet count ×mean platelet volume, 0.19). Finally, the activities of other plasma coagulation factors were normal or above normal (fibrinogen and factors V, VIII, IX, and factor XI) but the activities of coagulation factor VII were low (1.8%). Therefore, by process of elimination, our diagnosis was FVII deficiency.

To determine whether our patient's FVII deficiency was acquired or congenital, we used samples of his peripheral blood for second‐generation sequencing: 86 genes related to thrombosis and hemostasis (Supplementary file: Table 1) were sequenced on an Illumina Miseq Next‐Generation Sequencer, including hot spot mutations (CNV and InDel), variable splicing sites and large fragment deletion mutations in gene coding regions and intron sequences of 20bp regions upstream and downstream of exon. The average sequencing depth was 500‐folds, and the genome coverage rate was 99.47% (Supplementary file: Table 2). Indicative of a congenital origin, two heterozygous mutations in the *F7* gene, which encodes FVII, were identified: c. 681+1 G>T in intron 7 and c. C1286T (p. Ala429Val) in exon 9.

As treatment, we administered fresh frozen plasma (about 800 ml/day on average) for 11 days after admission, resulting in a relatively good PT (19.8–24.4 s). He also twice received fresh frozen plasma in combination with rFVIIa, and on the 19th day of admission, rFVIIa alone. The total dose of rFVIIa was 15 mg; for economic reasons, rFVIIa was only administered three times. The PT was maintained at 22.3–24.8 s during the combined treatment. After 2 months of therapy, he was discharged uneventfully.

## 3 DISCUSSION

rFVIIa is considered the most effective treatment for congenital FVII deficiency with severe bleeding, but has three main disadvantages: (1) it requires frequent bolus injections to offset its short half‐life, (2) it is difficult to obtain, and (3) it is extremely expensive. Fresh frozen plasma also has a short life and requires frequent injections; however, it is cheap and easily available and can be used in large quantities over time to maintain a reasonable PT value.

A literature search of *F7* mutations uncovered new missense mutations in only 33 of 140 cases of congenital FVII deficiency; among the 140 cases, more than 70 were reported in the past 10 years. Relevant information is summarized in Table 2.

**TABLE 2:** Cases of congenital FVII deficiency reported in the past decade: Novel missense mutation of the FVII gene.

Mutants in the FVII gene	
Missense	Reference
p. Met1Thr	Rath (2015) *Hamostaseologie* 35S1, S36
p. Gln16Term	Rath (2015) *Hamostaseologie* 35S1, S36
p. Gly22Cys	Shigekiyo (2015) *Blood Coagul Fibrinolysis* 26, 956
p. Cys82Gly	Shigekiyo (2015) *Blood Coagul Fibrinolysis* 26, 956
p. Cys82Tyr	Borhany (2013) *Haemophilia* 19, 893
p. Ser127Pro	Rath (2015) *Hamostaseologie* 35S1, S36
p. Gln160Leu	Jin (2012) *Zhonghua Yi Xue Yi Chuan Xue Za Zhi* 29, 404
p. Ser190Phe	Jiang (2011) *Blood Coagul Fibrinolysis* 22, 264
p. Ala191Thr	Sakakibara (2015) *Pediatr Int* 57, 1023
p. Trp247Leu	Rath (2015) *Hamostaseologie* 35S1, S36
p. Ser250Phe	Jiang (2011) *Blood Coagul Fibrinolysis* 22, 264
p. Glu270Lys	Rath (2015) *Hamostaseologie* 35S1, S36
p. Arg277Cys	Hao (2015) *Blood Coagul Fibrinolysis* 26, 687
p. Tyr294Term	Suzuki (2013) *Thromb Res* 131, 166
p. Pro311Leu	Mourey (2014) *Haemophilia* 20, e347
p. Leu314Val	Kwon (2011) *Blood Coagul Fibrinolysis* 22, 102
p. Pro320Leu	Kogiso (2011) *Clin Appl Thromb Hemost* 17, E205
p. Cys322Ser	Borhany (2013) *Haemophilia* 19, 893
p. Ser329Pro	Riccardi (2012) *Haemophilia* 18S3, 187
p. Trp344Gly	Hao (2016) *Blood Coagul Fibrinolysis* 27, 461
p. Leu357Phe	Borhany (2013) *Haemophilia* 19, 893
p. Asn361Ile	Giansily‐Blaizot (2016) *Haemophilia* 22, e304
p. Phe388Tyr	Elmahmoudi (2012) *Diagn Pathol* 7, 92
p. Cys389Tyr	Rath (2015) *Hamostaseologie* 35S1, S36
p. Thr410Ala	Borhany (2013) *Haemophilia* 19, 893
p. Tyr412Cys	Rath (2015) *Hamostaseologie* 35S1, S36
p. Gln426Term	Jiang (2011) *Zhonghua Xue Ye Xue Za Zhi* 32, 147
p. Tyr443Cys	Krouwel (2013) *Haemophilia* epub, epub
p. Arg353Gln	Jin (2018) *Blood Coagul Fibrinolysis* 67, 74
p. Term467Gln	Giansily‐Blaizot (2016) *Haemophilia* 22, e304
p. Leu−48Pro and p. Pro260Leu	Kogiso (2011) *Clin Appl Thromb Hemost* 17, 205

Note

FVII, factor VII. References are listed by first author, year of publication and the journal.

According to the report, more than 200 mutations, mostly missense mutations, have been identified in the *F7* gene, which is located on chromosome 13.^6^ The diversity of the FVII genotype accounts for the multiple mechanisms underlying congenital FVII deficiency and the difficulty in diagnosing this condition. Our study identified two heterozygous mutations: c. 681+1 G>T in intron 7 and c. C1286T (p. Ala429Val) in exon 9. Without the whole‐genome sequencing, we could not determine whether there were other mutations in the undetected introns, but we did not find other mutations in the range we sequenced. The c. 681+1 G>T mutation has been reported in six previous cases (Table 3) in the European Association for Haemophilia and Allied Disorders blood coagulation factor VII variant database (https://f7‐db.eahad.org/). In these cases, the most severe and common symptom was intracranial hemorrhage (2 cases), which was consistent with the patient we reported, and other manifestations included gastrointestinal bleeding (1 case), easy bruising (1 case), menorrhagia, and gum bleeding (1 case). Furthermore, the patients’ age in previous reports was almost less than 40 (3 days–38 years). However, we report a 66‐year‐old congenital FVII deficiency elder patient. Although Ala429 mutations have been reported before [e.g., Ala429Thr (c. G1285A)],^7^ the p. Ala429Val mutation is novel and thus further expands the diversity of the FVII genotype.

**TABLE 3:** Clinical data with c. 681+1 G>T mutation of F7 gene

Case ID	Gender	Age	Coagulation parameters			FVII:C%	Symptom	Severtity	Mutation	Genotype	Family history	Reference
			PT	APTT	TT							
783	Male	3 days	P	N	N	5	Intracranial bleeding	Severe	c.681+1G>T, c.431‐2A>G	Heterozygous	No significant bleeding	Ariffin H (2003) *J Pediatr Hematol Oncol* 25,418
754	Female	21	‐	‐	‐	‐	Intracranial bleeding	Severe	c.681+1G>T	Heterozygous	‐	Cavallari N (2012) *Biochim Biophys Acta* 1822,1109
	Male	7	‐	‐	‐	‐	Gastrointestinal bleeding	Severe	c.681+1G>T	Heterozygous	‐	
562	Female	10	‐	‐	‐	3	Easy bruising	Severe	c.681+1G>T, c.1027G>A	Heterozygous	‐	Herrmann FH (2009) *Haemophilia* 15,267
452	Female	38	P	N	N	2.1	Menorrhagiaand gum bleeding	Mild	c.681+1G>T, c.839A>C	Heterozygous	No significant bleeding	Liu H, (2015) *Blood Coagul Fibrinolysis* 26,408
315	‐	‐	‐	‐	‐	30	‐	Mild	c.681+1G>T	Heterozygous	‐	Peyvandi F (2000) *Thromb Haemost* 84,250
281	‐	‐	‐	‐	‐	45	‐	Mild	c.681+1G>T, c.1238G>A, c.−325_−324insCCTATATCCT	Heterozygous	‐	Millar DS (2000) *Hum Genet* 107,327

Abbreviations: N, Normal; P, Prolonged; References are listed by first author, year of publication and the journal.

**FIGURE 2:**
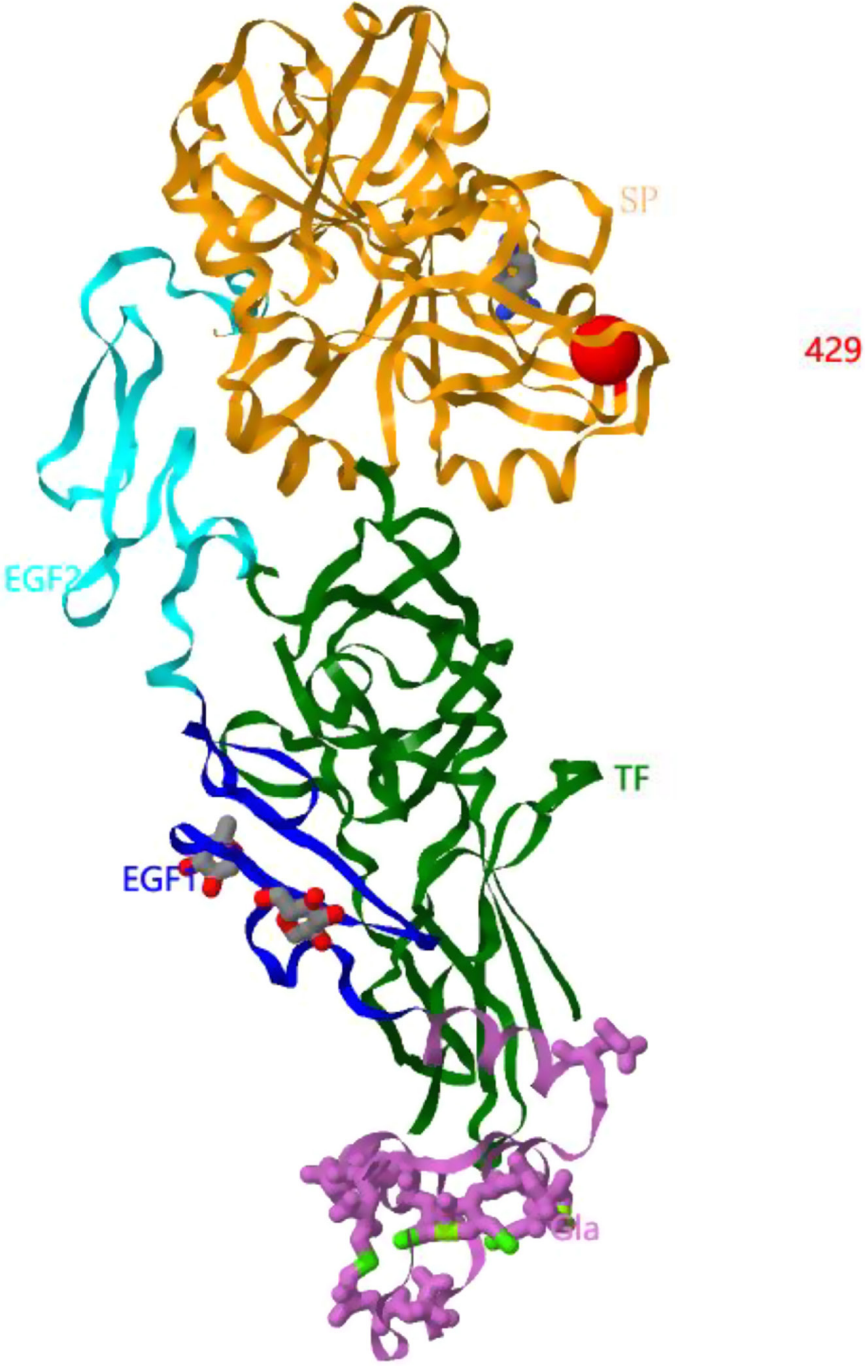
Predicted protein structure of FVII‐TF Complex. The predicted protein structure of FVII‐TF complex from factor VII gene (F7) variant database (https://f7‐db.eahad.org/novel.html.php). The red sphere is mutated V429 residue. The orange part is the serine protease (SP) domain of FVII. The dark blue part is the epidermal growth factor 1(EGF1) domain of FVII. The light blue part is the epidermal growth factor 2(EGF2) domain of FVII. The purple part is the gamma‐carboxyglutamic acid (Gla) domain of FVII. The green part is the tissue factor (TF) domain of FVII

We performed a structural prediction analysis of the FVII protein in our patient. The results showed that the variant Val429 residue was within a hydrophobic region, as is the native residue, and was buried in the serine protease domain (surface accessibility is 1) (Figure 2).

During the coagulation cascade, FVII needs to be activated before binding to an exposed tissue factor, thereby initiating the extrinsic coagulation pathway. Activation of FVII requires the participation of the serine protease domain and proteolytic enzymes.^8^ We suggest that the Ala429Val missense mutation changes the molecular spatial conformation of the serine protease domain, ultimately inhibiting the formation of FVII‐tissue factor complexes and the extrinsic coagulation pathway.

## 4 CONCLUSION

We identified a novel *F7* mutation, thus adding to the collection of variant human *F7* genes. The specific pathogenesis associated with this mutation requires further experimental investigation.

## CONFLICTS OF INTEREST

There are no conflicts of interest.

## AUTHOR CONTRIBUTIONS

Hua Tang contributed to conceptualization, software, and writing—original draft. Xingzhao Luan contributed to software and image curation. Jiaqi Li contributed to methodology and software. Gen Jiang contributed to data collection. Haowen Zhen and Hao Li contributed to supervision. Wei Xiang contributed to conceptualization, methodology, and revision. Jie Zhou contributed to supervision and writing—review and editing.

## CONSENT FOR PUBLICATION

Written informed consent was obtained from the family members of the patient.

## Supporting information

Supplementary Material

## Data Availability

All relevant data are included in the article.
