# Supplementary material for: Novel heterozygous F7 gene mutation (c. C1286T) associated with congenital factor VII deficiency: A case report and literature review
Source: J Clin Lab Anal. 2022 Mar 29;36(5):e24349. doi: 10.1002/jcla.24349 (PMC9102670; doi:10.1002/jcla.24349)
Supplement: Supplementary file 1 — Supplementary Material [file JCLA-36-e24349-s001.docx]

**Table 1. The List of sequenced 86 genes related to thrombosis and hemostasis**

| **Abbreviations** | **Gene name** |
| --- | --- |
| ABCB1 | ATP Binding Cassette Subfamily B Member 1 |
| ABCG2 | ATP Binding Cassette Subfamily G Member 2 |
| ADAMTS13 | ADAM Metallopeptidase With Thrombospondin Type 1 Motif 13 |
| ANKRD26 | Ankyrin Repeat Domain Containing 26 |
| AP3B1 | Adaptor Related Protein Complex 3 Subunit Beta 1 |
| BLOC1S3 | Biogenesis Of Lysosomal Organelles Complex 1 Subunit 3 |
| BLZF1 | Basic Leucine Zipper Nuclear Factor 1 |
| CES1 | Carboxylesterase 1 |
| CYCS | Cytochrome C, Somatic |
| CYP2C19 | Cytochrome P450 Family 2 Subfamily C Member 19 |
| CYP2C9 | Cytochrome P450 Family 2 Subfamily C Member 9 |
| CYP3A4 | Cytochrome P450 Family 3 Subfamily A Member 4 |
| CYP4F2 | Cytochrome P450 Family 4 Subfamily F Member 2 |
| DTNBP1 | Dystrobrevin Binding Protein 1 |
| F2 | Coagulation Factor II |
| F5 | Coagulation Factor V |
| F7 | Coagulation Factor VII |
| F8 | Coagulation Factor VIII |
| F9 | Coagulation Factor IV |
| F10 | Coagulation Factor X |
| F11 | Coagulation Factor XI |
| F13A1 | Coagulation Factor XIII A Chain |
| F13B | Coagulation Factor XIII B Chain |
| FGA | Fibrinogen Alpha Chain |
| FGB | Fibrinogen Beta Chain |
| FGG | Fibrinogen Gamma Chain |
| FLI1 | Fli-1 Proto-Oncogene, ETS Transcription Factor |
| FLNA | Filamin A |
| GATA1 | GATA Binding Protein 1 |
| GGCX | Gamma-Glutamyl Carboxylase |
| GP1BA | Glycoprotein Ib Platelet Subunit Alpha |
| GP1BB | Glycoprotein Ib Platelet Subunit Beta |
| GP6 | Glycoprotein VI Platelet |
| GP9 | Glycoprotein IX Platelet |
| HIVEP1 | HIVEP Zinc Finger 1 |
| HOXA11 | Homeobox A11 |
| HPS1 | HPS1 Biogenesis Of Lysosomal Organelles Complex 3 Subunit 1 |
| HPS3 | HPS3 Biogenesis Of Lysosomal Organelles Complex 2 Subunit 1 |
| HPS4 | HPS4 Biogenesis Of Lysosomal Organelles Complex 3 Subunit 2 |
| HPS5 | HPS5 Biogenesis Of Lysosomal Organelles Complex 2 Subunit 2 |
| HPS6 | HPS5 Biogenesis Of Lysosomal Organelles Complex 2 Subunit 3 |
| HRG | Histidine Rich Glycoprotein |
| ITGA2B | Integrin Subunit Alpha 2b |
| ITGB3 | Integrin Subunit Beta 3 |
| KLKB1 | Kallikrein B1 |
| KNG1 | Kininogen 1 |
| LMAN1 | Lectin, Mannose Binding 1 |
| LYST | Lysosomal Trafficking Regulator |
| MCFD2 | Multiple Coagulation Factor Deficiency 2, ER Cargo Receptor Complex Subunit |
| MPL | MPL Proto-Oncogene, Thrombopoietin Receptor |
| MTHFR | Methylenetetrahydrofolate Reductase |
| MYH9 | Myosin Heavy Chain 9 |
| NBEA | Neurobeachin |
| NBEAL2 | Neurobeachin Like 2 |
| NME7 | NME/NM23 Family Member 7 |
| P2RY12 | Purinergic Receptor P2Y12 |
| PEAR1 | Platelet Endothelial Aggregation Receptor 1 |
| PLA2G4A | Phospholipase A2 Group IVA |
| PLAT | Plasminogen Activator, Tissue Type |
| PLAU | Plasminogen Activator, Urokinase |
| PLG | Plasminogen |
| PROC | Protein C, Inactivator Of Coagulation Factors Va And VIIIa |
| PROCR | Protein C Receptor |
| PROS1 | Protein S |
| PROZ | Protein Z, Vitamin K Dependent Plasma Glycoprotein |
| PTGS2 | Prostaglandin-Endoperoxide Synthase 2 |
| RBM8A | RNA Binding Motif Protein 8A |
| RUNX1 | RUNX Family Transcription Factor 1 |
| SERPINA1 | Serpin Family A Member 1 |
| SERPINC1 | Serpin Family C Member 1 |
| SERPIND1 | Serpin Family D Member 1 |
| SERPINE1 | Serpin Family E Member 1 |
| SERPINF2 | Serpin Family F Member 2 |
| SLC44A2 | Solute Carrier Family 44 Member 2 |
| SMAP1 | Small ArfGAP 1 |
| TBXA2R | Thromboxane A2 Receptor |
| TBXAS1 | Thromboxane A Synthase 1 |
| TFPI | Tissue Factor Pathway Inhibitor |
| THBD | Thrombomodulin |
| THPO | Thrombopoietin |
| TSPAN15 | Tetraspanin 15 |
| TUBB1 | Tubulin Beta 1 Class VI |
| VKORC1 | Vitamin K Epoxide Reductase Complex Subunit 1 |
| VWF | Von Willebrand Factor |
| WAS | WASP Actin Nucleation Promoting Factor |
| ZFPM2 | Zinc Finger Protein, FOG Family Member 2 |

**Table 2. The Summary of NGS statistics result of the patient**

| **NGS statistics** | **Result** |
| --- | --- |
| Raw bases(Mb) | 1456.04 |
| Clean bases(Mb) | 1235.76 |
| QCrate(%) | 84.87 |
| Target average read length | 129 |
| Target average base quality | 36.5 |
| Target average insert size | 152.5 |
| Target duplicated bases(Mb) | 200.97 |
| Target duplication rate(%) | 20.70 |
| Total mapped reads(M) | 9.52 |
| Total reads mapping rate(%) | 99.41 |
| Target mapped reads(M) | 7.52 |
| Target reads capture rate(%) | 79.02 |
| Flank mapped reads(M) | NA |
| Flank reads capture rate(%) | NA |
| Target size | 617680 |
| Target covered size | 617501 |
| Coverage rate(%) | 99.97 |
| Target effective bases(Mb) | 563.82 |
| Target effective rate(%) | 58.87 |
| Target mean depth | 912.80 |
| T 4X coverage rate(%) | 99.90 |
| T 10X coverage rate(%) | 99.86 |
| T 20X coverage rate(%) | 99.81 |
| T 30X coverage rate(%) | 99.75 |
| T 10%X coverage rate(%) | 99.23 |
| T 20%X coverage rate(%) | 98.42 |
| T 30%X coverage rate(%) | 96.99 |
| T 50%X coverage rate(%) | 91.21 |
| T Mean depth flank 10%X coverage rate(%) | 22.02 |
| Panel name | T367V2 |
